# Nearly complete genome sequence of a *Perhabdovirus* isolated on European perch (*Perca fluviatili*s)

**DOI:** 10.1128/MRA.00442-23

**Published:** 2023-09-22

**Authors:** Laurane Pallandre, Doriana Flores, Françoise Pozet, Laurent Bigarré

**Affiliations:** 1 Laboratoire de Ploufragan-Plouzané-Niort, ANSES, technopole Brest-Iroise, Plouzané, France; 2 Jura Departemental Analysis Laboratory, Poligny, France; Katholieke Universiteit Leuven, Leuven, Belgium

**Keywords:** *Perhabdovirus*, fish, pathogen, MinION, genome

## Abstract

We report a nearly full-length genome of a *Perhabdovirus* isolated in 2022 on perch on a French farm. This virus is genetically related to virus 20/43, which was associated with an outbreak of perch on a farm in France in 2019. Both viruses represent a specific lineage of perch rhabdovirus.

## ANNOUNCEMENT

*Perhabdovirus* (family *Rhabdoviridae*) is a genus that includes three viral species, all of which infect fish (1). One species, namely *Perhabdovirus perca*, is represented by a number of viral isolates, including perch rhabdovirus (2–6). We report a nearly complete genomic sequence of a *Perhabdovirus* (22/145) isolated in France during a mortality event of farmed European perch in July 2022. This virus is genetically closely related to virus 20/43 previously identified on another farm in the same region in 2019 (7).

Organs (kidney, spleen, and brain) of sick perch (*Perca fluviatilis*) were homogenized and inoculated with a culture of BF2 cells. After a few days, a typical cytopathic effect appeared, suggesting the multiplication of a virus, presumably a rhabdovirus. Nucleic acids were extracted from the culture supernatant using a virus nucleospin kit (Macherey-Nagel) and submitted to RT-PCR targeting a large portion of the glycoprotein gene of all *Perhabdoviruses* (8). A volume of 5 μL of nucleic acids was used for amplification with the superscript III kit (Thermofisher). The result was the production of a single amplicon of 1,330 bp, as expected for a *Perhabdovirus*. Its sequence, obtained by the Sanger method, exhibited the highest nucleic acid similarity (97.4%) with the homologous sequence of isolate 20/43 originating from perch farmed in another site in the same region.

**Fig 1:**
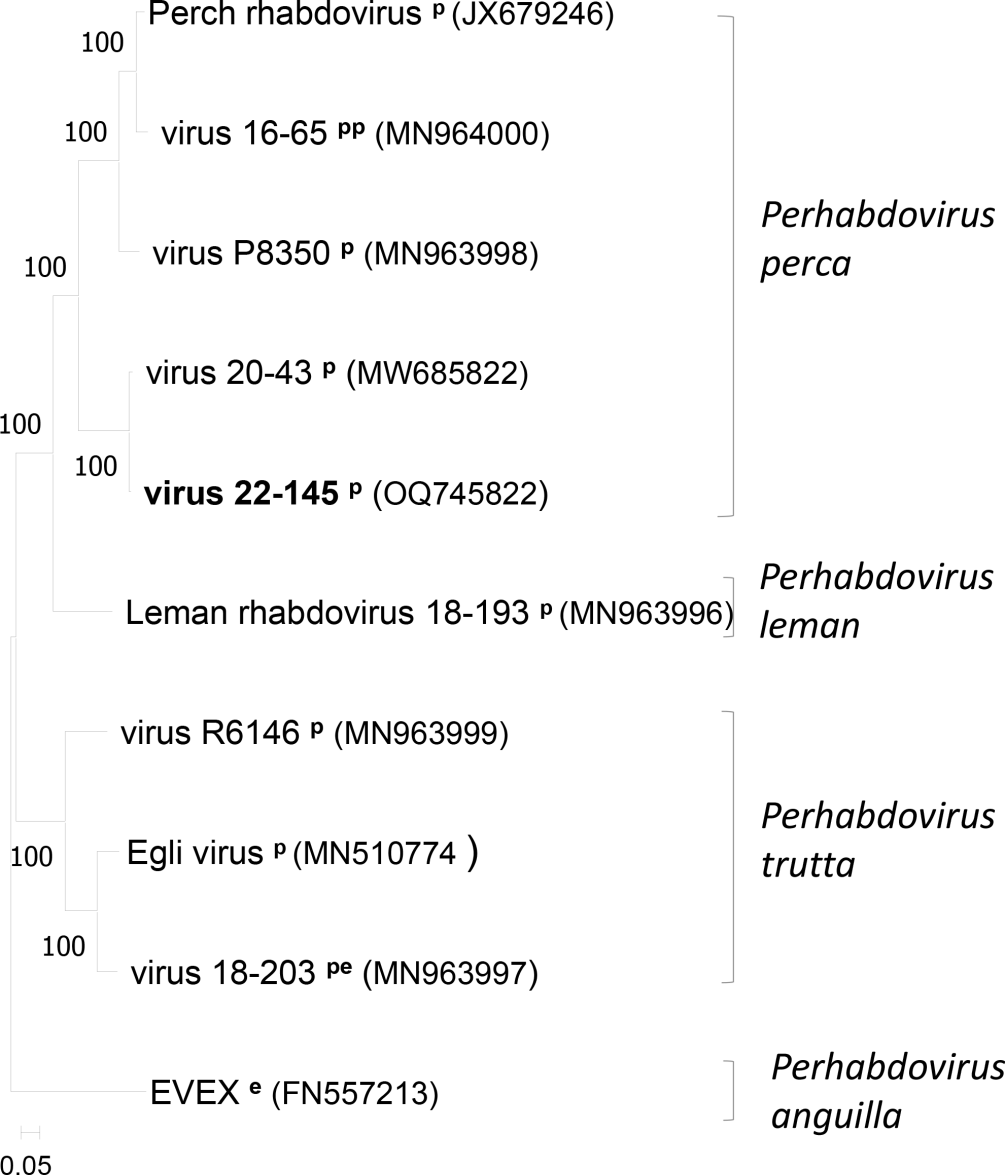
Phylogeny of concatenated ORFs of *Perhabdoviruses*. The alignment was performed with the clustal method of MEGA11 (9), and the phylogeny was obtained using the maximum-likelihood method (1,000 replicates). Eel virus X (EVEX) is used as an outgroup. The scale bar indicates genetic distance (substitutions/site). For each virus, the host is indicated as an abbreviation after the name: p, European perch; pe, percidae; e, eel; pp, pike-perch.

This resemblance was used to obtain the nearly complete sequence of isolate 22/145 by producing and sequencing PCR tiled amplicons covering the genome. Based on the sequence of isolate 20/43 (MW685822), four sets of primers were designed and proved to be efficient in amplifying tiled genomic portions of virus 22/145 (Table 1). After purification, the four amplicons were dosed and pooled in equal weight (60 ng/amplicon) to produce a library using the ligation sequencing kit DNA V14 (Oxford Nanopore technology). Sequencing was performed with a flongle (ONT) for 22 h followed by fast basecalling. The reads were aligned to the reference 20/43 using GENEIOUS prime (2023.0.4, Biomatters). Of 213k reads, a total of 206k reads were aligned to the reference sequence. A consensus sequence of 11,380 nt (GC content of 43.5%), excluding the primers at the extremities, was obtained. The assembled genome of virus 22/145 covered 99.5% of the genome of 20/43 (11,434 nt). In total, 19 positions were ambiguous, showing two possible nucleotides at frequencies superior to 26%, far higher than the announced error rate of the sequencing technology (1%). When two nucleotides were proposed at the same position, the one with the highest frequency was chosen. Aligned to the genome of 20/43 using the clustal omega function of GENEIOUS, the sequence of 22/145 differed by 158 single nucleotide polymorphisms (SNPs) (identity 98.6%). The open reading frame (ORF) composition was identical to the one of virus 20/43 (mucleoprotein-phosphoprotein-matrix protein-glycoprotein-RNA polymerase), and the two sequences of the concatenated ORFs were the most related phylogenetically (Fig. 1). Together with virus 20/43, virus 22/145 is the second virus belonging to a specific lineage genetically distinct from the one represented by Perch rhabdovirus.

**TABLE 1:** Features of the primers used for producing the amplicons of virus 22/145^*a*^

Sets of primers	Primer sequence (5′−3′)	Size of amplicon (bp)
oPVP909oPVP910	CTTAATGAACTGAATTTTTCTCTGG GCGCAGGCTAGATGTATAGTTC	3,124
oPVP911oPVP912	GGCTAGCGTGAGAGGATCTC CTGTTATTCAGAGCTTCTTCAGC	3,079
oPVP913oPVP914	CAGAGATTGCCAGGACATACC ACCATCCTGTGGTCGTCGG	3,096
oPVP915oPVP916	ACCATTTTCACCTTTCCTGTG ACCACACCAGATCTCTTTTACAC	2,494
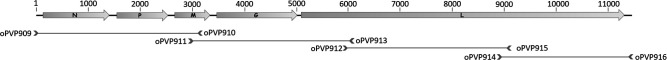		

^*a*^ The positions of the primers are indicated on the genetic map of virus 20/43, the most genetically related to 22/145.

## Data Availability

The genomic sequence of virus 22/145 has been deposited in Genbank under the accession number OQ745822. The reads are available at the SRA database under the reference PRJNA992259.
